# Supplementary material for: A High-Throughput Small-Angle X-ray Scattering Assay to Determine the Conformational Change of Plasminogen
Source: Int J Mol Sci. 2023 Sep 19;24(18):14258. doi: 10.3390/ijms241814258 (PMC10531915; doi:10.3390/ijms241814258)
Supplement: Supplementary file 1 [file ijms-24-14258-s001.zip › ijms-2581903-supplementary.pdf]

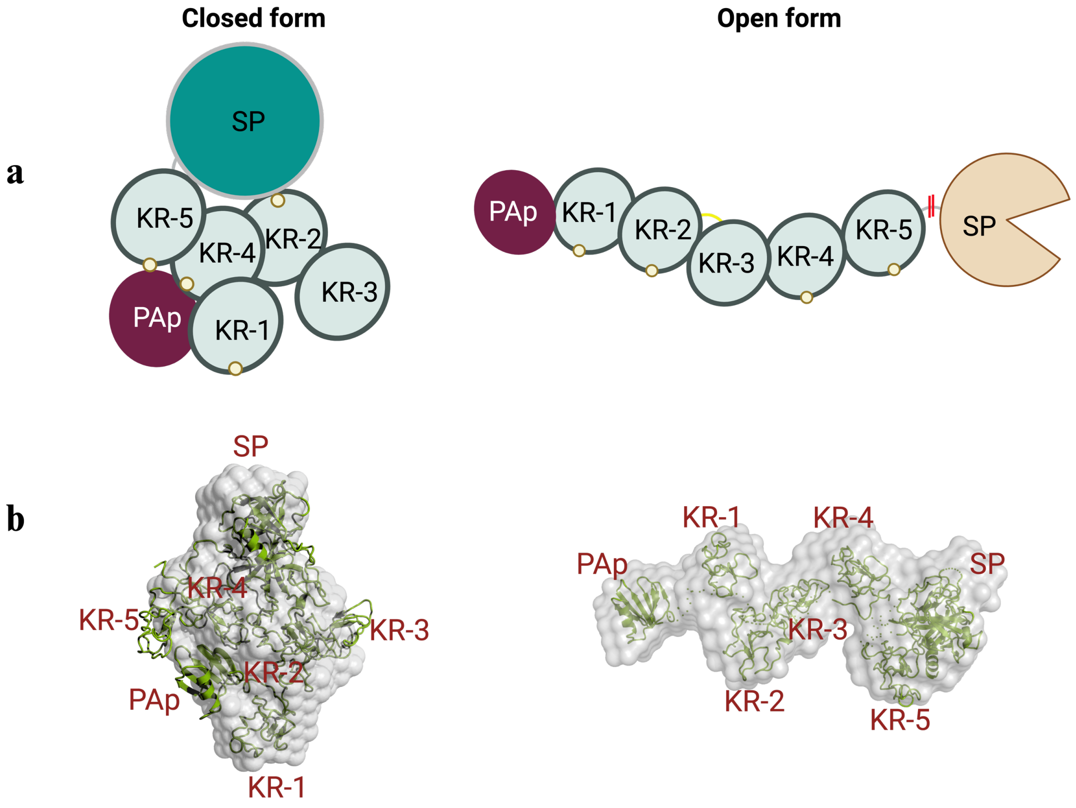

Closed form
Open form
a
SP
KR-5
KR-4
KR-2
KR-3
PAp
KR-1
PAp
KR-1
KR-2
KR-3
KR-4
KR-5
SP
b
SP
KR-4
KR-5
KR-3
KR-2
PAp
KR-1
KR-1
KR-4
PAp
SP
KR-3
KR-2
KR-5

c

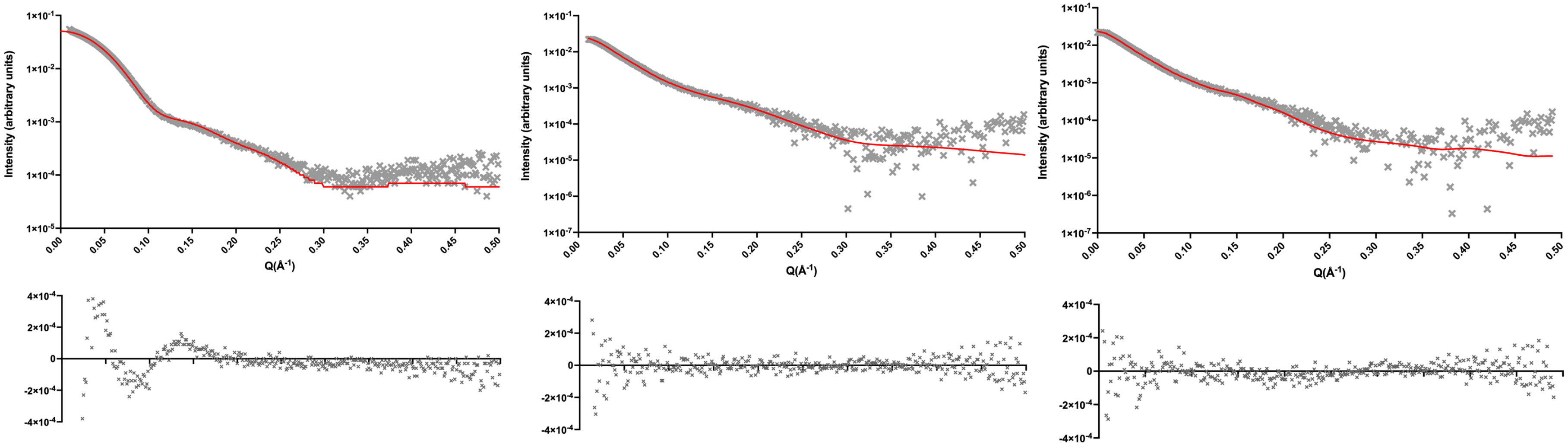

Intensity (arbitrary units)
Q(Å⁻¹)
1×10⁻¹
1×10⁻²
1×10⁻³
1×10⁻⁴
1×10⁻⁵
1×10⁻⁶
1×10⁻⁷
0.00
0.05
0.10
0.15
0.20
0.25
0.30
0.35
0.40
0.45
0.50
4×10⁻⁴
2×10⁻⁴
0
-2×10⁻⁴
-4×10⁻⁴

d

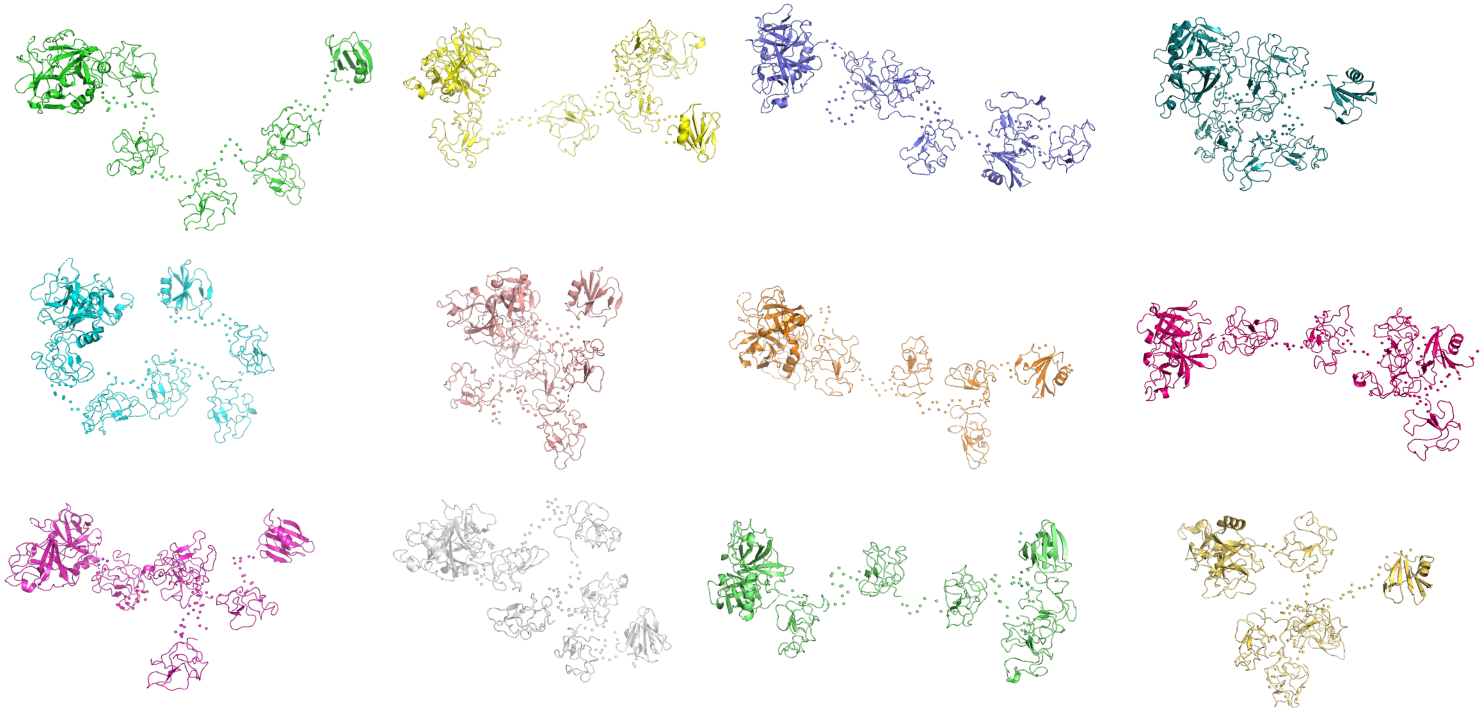

Figure S1. Cartoon representation of closed and open Plg**.** Plg is a 7-domain zymogen with an N-terminal PAN-domain (PAp), five kringle domains (KR1-5), and a serine protease domain (SP). In the closed conformation (**a**), the lysine binding site (D/EXD motif, LBS) found on KR-2, 4 and 5 (yellow circle) binds to SP, PAp and PAp domain, respectively; LBS in KR-1 is free. Here, the activation loop (grey line) is not exposed. The amino acid sequence corresponding to the KR-3 LBS is DXK, which does not bind to lysine or arginine residues. Open Plg forms through an interaction between the LBSs and free lysine/analogues or surface lysine/arginine on target sites; it also exposes the activation loop, allowing activation by plasminogen activators. This illustration is generated using BioRender (Agreement number: PY25V3WN5F). (**b**) A side-by-side comparison of the *ab initio* low-resolution envelope models of the representative structure of closed and open Plg GII. Also shown is a superposition of the X-ray crystal structure of closed Plg GII as detailed in Figure 5 and the manuscript (left). Superposition of the most representative open Plg generated using ATSAS program BUNCH is also shown (right). (**c**) SAXS data are shown as grey crosses with red lines representing the fit to structural models. On the left: the fit using CRYSOL of the crystal structure shown in **panel b** above to the SAXS data of closed/unliganded Plg. In the middle: fit of the most representative

model from five repeats of BUNCH shown on the right of **panel b** above to SAXS data of the open/liganded Plg. On the right: Fit of the best ensemble of structures from EOM shown in **panel b** above to the open Plg. (**d**) 12 Representative models of open Plg from EOM analysis where the seven domains are connected via flexible interdomain linkers except for KR2 and KR3, which are constrained by an interdomain disulphide linkage.

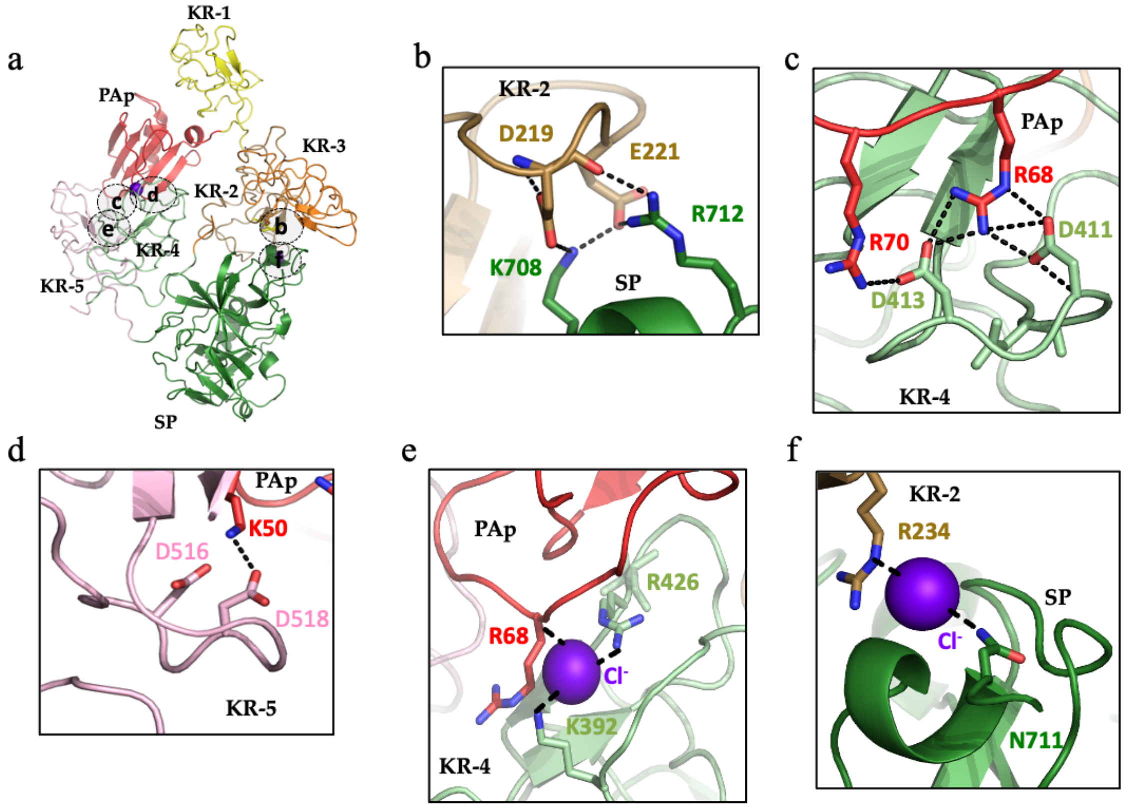

Figure S2. Interdomain interactions in closed Plg GII (a) Cartoon representation of Plg GII X-ray crystal structure. Letters enclosed in dotted circles represent regions involved in inter-domain interactions shown in the corresponding panels b-f. (b) Inter-domain interactions between KR-2 LBS and SP domain. (c) Inter-domain interactions between KR-4 LBS and PAp domain. (d) Inter-domain interactions between KR-5 LBS and PAp domain. (e-f) Chloride (Cl-) ions (depicted as purple spheres) also mediate inter-domain interactions between KR-2/SP and KR-4/PAp.

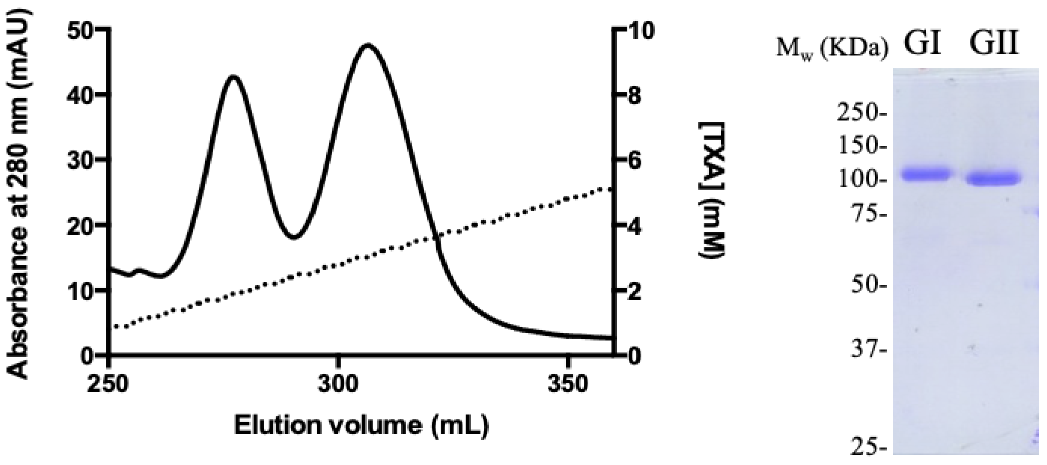

Figure S3. Left panel: Chromatogram showing the separation of Plg GI and GII on Lysine Hyper D Resin. **Right panel:** SDS-PAGE showing purified glycoforms.

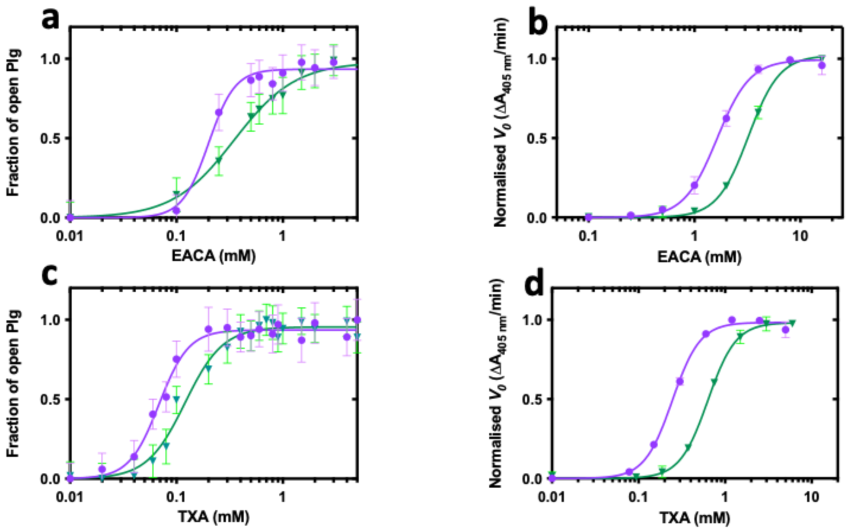

Ligand	K_{open} (mM)		$K_{activation}$ (mM)	
	Plg GI	Plg GII	Plg GI	Plg GII
EACA	0.20 ± 0.01	0.35 ± 0.02	1.63 ± 0.05	3.23 ± 0.06
TXA	0.068 ± 0.002	0.120 ± 0.008	0.25 ± 0.01	0.63 ± 0.01

Figure S4. Comparison of ligand-induced conformational change and tPA activation of Plg glycoforms. Experimental data presented in **Table 1** on the conformational change of Plg GI (purple) and GII (green) are shown (**a**) EACA and (**c**) TXA. K_{open} is the ligand concentration required to induce the open conformation in 50% of the total Plg in solution. TXA exhibited lower K_{open} (higher efficacy) for both glycoforms than EACA (**Table below**). tPA is used to determine the rate of Plg activation in the presence of EACA (**b**) and TXA (**d**). Normalized initial rates are plotted against their respective ligand concentration. $K_{activation}$ is the ligand concentration at which the initial velocity is 50% of the maximal rate. The $K_{activation}$ of TXA is also lower than that of EACA. Compared with K_{open}, the $K_{activation}$ concentration is significantly higher ($P < 0.0001$), >8-fold for EACA, and >3.5-fold for TXA, suggesting Plg activation by tPA may occur only on the fully open molecules.

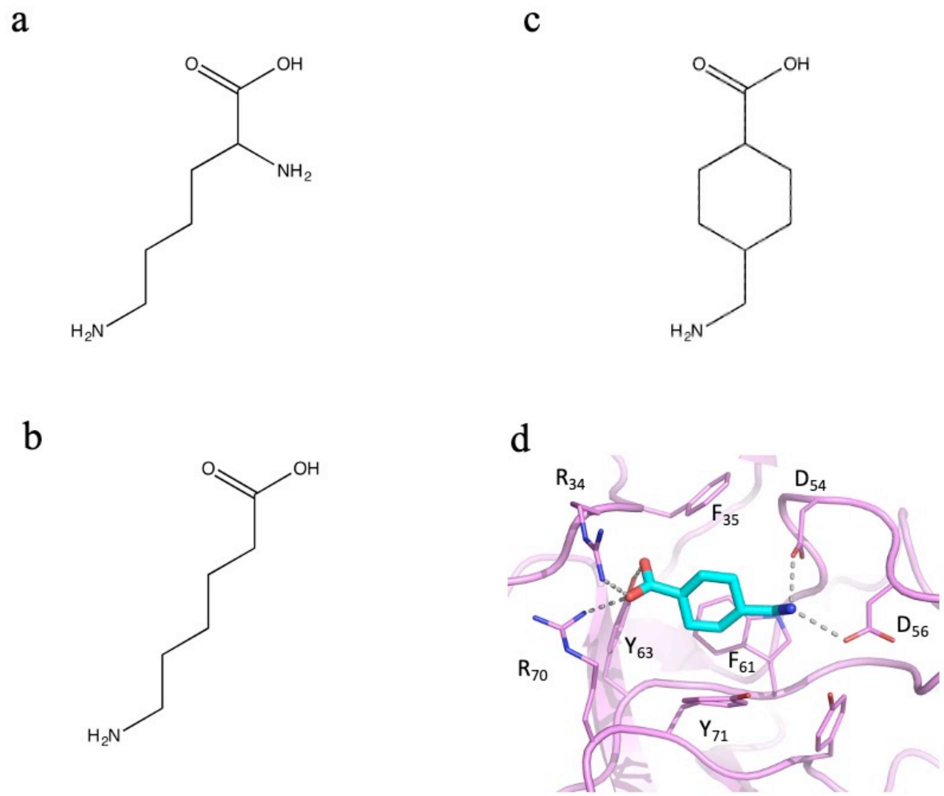

Figure S5. Lysine and analogues and molecular interaction with lysine binding site of KR-1**.** Chemical structures of (**a**) lysine, (**b**) ε-aminocaproic acid (EACA) and (**c**) tranexamic acid (TXA). **(d)** Cartoon representation of the co-crystal structure of KR-1(light purple) and TXA (cyan) (PDB ID 1CEB) [1], where residues bind to TXA in the LBS, are labelled and shown in dotted lines.

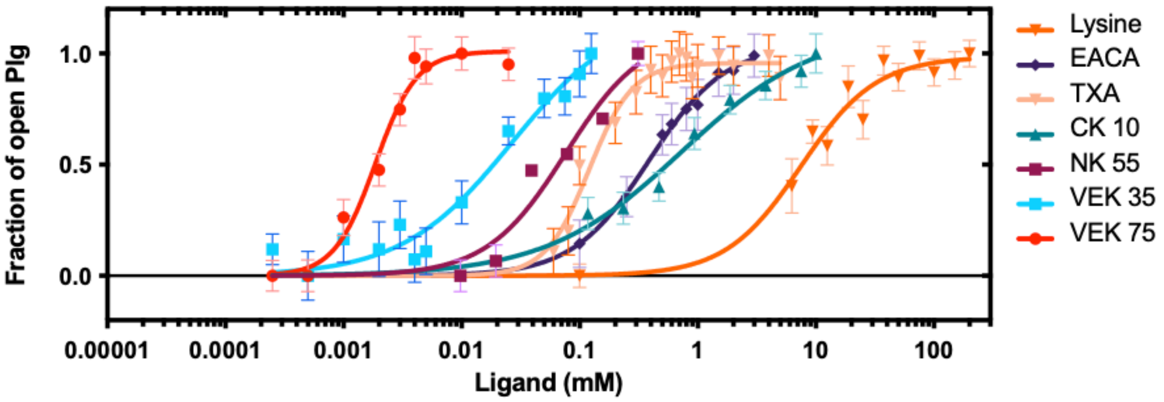

Ligand/peptide	K_{open} (mM)
L-Lysine	7.47 ± 1.12
EACA	0.35 ± 0.02
TXA	0.120 ± 0.008
CK10	0.73 ± 0.24
MK12	*ND**
NK55	0.076 ± 0.046
VEK35[a]	***0.027 ± 0.016***
VEK75[a]	***0.0019 ± 0.00018***

Figure S6. Titration curves of ligands and peptides showing a concentration-dependent conformational change of Plg GII. The K_{open} determined is summarized in the Table. The order of ligand efficacy is MK12>L-lysine>CK10>EACA>TXA>NK55. Also shown is the side-by-side comparison with our previous work on VEK peptides derived from the plasminogen binding Group A Streptococcal M protein PAM, VEK35 (GSVE***K***LTADAELQ***R***L***K***NE***R***HEEAELE***R***L***K***SE***R***HDHDY) and VEK75 (GSEELQGL***K***DDVE***K***LTADAELQ***R***L***K***NE***R***HEEAELE***R***L***K***SE***R***HDHD***KK***EAE***RK***ALED***K***LAD***K***QEHLNGAL***R***YINE***K***EA). PAM has a much higher

affinity for human Plg than the host receptors. The K_{open} for NK55 is 2.8-fold of VEK35 and 40-fold of VEK75. [a] refer to [2]. for details. ND, not determined.

References

1. Mathews, I.I.; Vanderhoff-Hanaver, P.; Castellino, F.J.; Tulinsky, A. Crystal structures of the recombinant kringle 1 domain of human plasminogen in complexes with the ligands epsilon-aminocaproic acid and trans-4-(aminomethyl)cyclohexane-1-carboxylic Acid. *Biochemistry* **1996**, *35*, 2567–2576.

2. Bhattacharya, S.; Liang, Z.; Quek, A.J.; Ploplis, V.A.; Law, R.; Castellino, F.J. Dimerization is not a determining factor for functional high affinity human plasminogen binding by the group A streptococcal virulence factor PAM and is mediated by specific residues within the PAM a1a2 domain. *J. Biol. Chem.* **2014**, *289*, 21684–21693. https://doi.org/10.1074/jbc.M114.570218.
